# Editorial: “You shall not pass” or “Let`s make a deal” - crosstalk between helminths and the host immune system

**DOI:** 10.3389/fcimb.2023.1244429

**Published:** 2023-07-14

**Authors:** Piotr Bąska, Irma Schabussova, Anna Zawistowska-Deniziak

**Affiliations:** ^1^ Division of Pharmacology and Toxicology, Department of Preclinical Sciences, Institute of Veterinary Medicine, Warsaw University of Life Sciences, Warsaw, Poland; ^2^ Institute of Specific Prophylaxis and Tropical Medicine, Medical University of Vienna, Vienna, Austria; ^3^ Department of Immunology, Institute of Functional Biology and Ecology, Faculty of Biology, University of Warsaw, Warsaw, Poland

**Keywords:** immune response, helminths, Schistosoma japomicum, Echinococcus granulosus, Haemonchus contortus

Parasites are multicellular organisms that infect both humans and animals. They have co-evolved with their hosts over millions of years. This co-evolution can be viewed as an “arms race” in which both sides have developed sophisticated attack and defense mechanisms. “Classical” immune system defense mechanisms such as phagocytosis or induced apoptosis are efficient against bacteria and viruses but usually fail to terminate a parasitic worm infection. This inefficiency has forced the development of a higher-level cooperation between immune, nervous, and endocrine systems. Through this network, gastrointestinal helminths can be expelled by increased muscle contractility and enhanced water release to gut, resulting in diarrhea (Bąska and Norbury, 2022). In some cases, however, it is more beneficial to allow the infection to persist rather than to develop a strong but harmful immune response. Surprisingly, this phenomenon is not only a necessary disadvantage to prevent immunopathology, but may also be beneficial to the host by alleviating symptoms of allergies and autoimmune diseases. The host-parasite interaction is extremely complicated (Gazzinelli-Guimaraes and Nutman, 2018), and depends on the species and genetic background of both the host (Klementowicz et al., 2012) and the parasite (Bąska et al., 2017) and therefore this subject was the focus of this Research Topic.

There is ample evidence that helminth infections may be beneficial for people suffering from allergies or autoimmune diseases. This topic was explored by Hou et al. and Gao et al. In both studies, molecules derived from *Schistosoma japonicum* were tested for their ability to alleviate the symptoms of colitis or allergic rhinitis in mouse models. Administration of attenuated *S. japonicum* eggs improved the severity of colitis in mice (Hou et al.), and treatment with a small molecule peptide of *S. japonicum*, SJMHE1, reduced the clinical symptoms of allergic rhinitis and suppressed the recruitment of inflammatory cells and eosinophils to the nasal mucosa, thereby reducing the immune response. (Gao et al.). The results are consistent with the literature suggesting the potential of certain parasites to dampen inflammation (Schabussova and Wiedermann, 2014; Długosz et al., 2019; Zawistowska-Deniziak et al., 2021; Zawistowska-Deniziak et al., 2022; and Schabussova et al., 2013). In studies by Hou et al. and Gao et al., improvement in disease symptoms was associated with an increase in regulatory responses. For example, SJMHE1 resulted in increased levels of IL-10 in serum and also increased numbers of regulatory B cells in the spleen (Gao et al.). Along these lines, attenuated *S. japonicum* eggs increased the levels of IL-10 and TGF-β1 and restored the Th_reg_/Th_17_ balance (Hou et al.). Furthermore, this modulation was associated with suppression of the glycolysis pathway and lipogenesis, as well as boosting fatty acid oxidation, showing the importance of energy metabolism in alleviating the symptoms of autoimmune diseases (Hou et al.). The Gao et al. and Hou et al. studies demonstrate that parasites have the potential to regulate host immune responses and highlight the complexity of interplay between host and parasite. The complexity of these interactions was also explored by Hou et al., who investigated the role of CD4^+^ and CD8^+^ T cells in the course of *Echinococcus granulosus* infection in a mouse model and their distribution in infected human patients. The study showed the number of CD4^+^ T cells was more abundant around cysts. Moreover, their role was confirmed in mice deficient in CD4 T cells, which had higher numbers of cysts compared with wild-type mice. Another aspect related to helminth infections – collagen deposition and distribution of α-Sma positive cells, has also been studied in terms of the time course of infection and parasite stage. The studies by Gao et al., Hou et al., and Hou et al. provide new insights into the immune response triggered by parasites and also provide new evidence for the possibility of using parasites or their molecules as cures for allergic and autoimmune diseases. However, deliberate infections of humans can be dangerous and lead to severe complications, so further studies of parasite-derived molecules are needed.

Most studies of parasite-induced modulation have been performed with laboratory parasite strains that are likely to have low genetic diversity. Previous studies have confirmed that immune responses can vary depending on the parasite isolate (Bąska et al., 2017). Liu et al. showed that different *Haemonchus contortus* isolates exhibit different patterns in protein expression. Although the authors did not focus on the interaction of specific isolates with host immune responses, their results support the hypothesis that due to differences in protein expression, their effects on host immune responses and metabolism may differ.

The articles published in the Research Topic “*You shall not pass or Let`s make a deal* - Crosstalk *between helminths and the host immune system*” examined the interactions of *E. granulosus* with the host response (Hou et al.), the potential of using antigens from *S. japonicum* to alleviate symptoms of colitis (Hou et al.) and allergic rhinitis (Gao et al.), and the proteomics of *H. contortus* (Liu et al.). The contributions shed light on the intricate interactions between parasites and hosts, and we hope that the results will inspire other scientists to explore the role of parasitology in basic and applied research.

## Author contributions

PB and IS wrote the MS. AZ-D reviewed manuscript. All authors contributed to the article and approved the submitted version.
